# Antioxidant, genotoxic and antigenotoxic activities of daphne gnidium leaf extracts

**DOI:** 10.1186/1472-6882-12-153

**Published:** 2012-09-13

**Authors:** Fadwa Chaabane, Jihed Boubaker, Amira Loussaif, Aicha Neffati, Somaya Kilani-Jaziri, Kamel Ghedira, Leila Chekir-Ghedira

**Affiliations:** 1 Unité de Pharmacognosie/Biologie Moléculaire 99/UR/07-03, Faculté de Pharmacie de Monastir, Monastir, 5000, Tunisie; 2 Laboratoire de Biologie Cellulaire et Moléculaire, Faculté de Médicine Dentaire, de Monastir, Rue Avicenne, 5000, Tunisie

**Keywords:** *Daphne gnidium*, Antioxidant, Antigenotoxic

## Abstract

**Background:**

Plants play a significant role in maintaining human health and improving the quality of human life. They serve humans well as valuable components of food, as well as in cosmetics, dyes, and medicines. In fact, many plant extracts prepared from plants have been shown to exert biological activity *in vitro* and *in vivo*. The present study explored antioxidant and antigenotoxic effects of *Daphne gnidium* leaf extracts.

**Methods:**

The genotoxic potential of petroleum ether, chloroform, ethyl acetate, methanol and total oligomer flavonoid (TOF) enriched extracts from leaves of *Daphne gnidium*, was assessed using *Escherichia coli* PQ37. Likewise, the antigenotoxicity of the same extracts was tested using the “SOS chromotest test”. Antioxidant activities were studied using non enzymatic and enzymatic method: NBT/Riboflavine and xantine oxidase.

**Results:**

None of the different extracts produced a genotoxic effect, except TOF extract at the lowest tested dose. Our results showed that *D. gnidium* leaf extracts possess an antigenotoxic effect against the nitrofurantoin a mutagen of reference. Ethyl acetate and TOF extracts were the most effective in inhibiting xanthine oxidase activity. While, methanol extract was the most potent superoxide scavenger when tested with the NBT/Riboflavine assay.

**Conclusions:**

The present study has demonstrated that *D. gnidium* leaf extract possess antioxidant and antigenotoxic effects. These activities could be ascribed to compounds like polyphenols and flavonoid. Further studies are required to isolate the active molecules.

## Background

The use of medicinal plants has always been part of human culture. The World Health Organization estimates that up to 80% of the world’s population relies on traditional medicinal system for some aspect of primary health care [1]. In some countries, governments encourage the use of indigenous forms of medicine rather than expensive imported drugs [2].

Extracts from plants have been reported to be effective in treating febrile illnesses, sleeping sickness, wounds, diarrhoea, reproductive and liver problems, circulatory and respiratory problems and parasitic infections [3-5]. Recently, there has been a great increase of interest in natural antioxidant of plant origin since they are viewed as promising therapeutic agents for free radical pathologies and also found to be useful as nutraceuticals due to their impact on the status of human health and disease prevention [6,7].

The protection afforded by plants has been attributed to various phenolic compounds which are increasingly becoming of interest in the food industry because they retard oxidative degradation of lipids and thereby improve food quality [8].

Reactive oxygen species (ROS), are known to cause the oxidation of biomolecules leading to cellular damage. The tissue injury caused by ROS may include DNA and protein damages, and oxidation of important enzymes. These events could consequently lead to the occurrence of various free radical-related diseases. In the human body, the toxic effects of ROS are combated regularly by a number of endogenous defence and protective mechanisms which include various enzymes and non-enzymatic antioxidants. These self-defence systems may also be supported by antioxidative compounds taken as foods, cosmetics and herb medicine [8].

It is also important to note that most of the traditional medicinal plants have never been the subject of exhaustive toxicological tests such as is required for modern pharmaceutical compounds. Based on their traditional use for long periods of time they are often assumed to be safe. However, research has shown that a lot of plants which are used as food ingredients or in traditional medicine have *in vitro* mutagenic [9-11] or toxic and carcinogenic [12] properties.

At present, there are several antigenotoxicity assays available, which include the micronucleus test, somatic mutation and recombination test (SMART), sister chromatid exchange (SCE) assay and the single cell gel electrophoresis (SCGE) or comet assay. The above-mentioned assays may involve a longer analysis time, a high cost, and specialized skill or may require addition of expensive reagents. Therefore, short-term bacterial assay: SOS chromotest assay is useful and gives an estimation of the genotoxic/antigenotoxic potential of substances [13].

*Daphne gnidium* is an evergreen shrub that grows in the Mediterranean area and can grow to a height of 2 m [12]. In folk medicine the infusion of the leaves is used as hypoglycemic [14] and to treat skin diseases [15,16]. This plant is also used in traditional textile [17] dyeing.

However, *Daphne gnidium* is considered dangerous because of its high toxicity. It produces headache, shivering, paleness, pupil dilatation, mouth and lips swelling, difficulty of deglutition, diarrhea and digestive spasms, convulsion, pulmonary disorders and death [15,18]. Accordingly, in the present study, the antioxidant properties of *Daphne gnidium* leaf extracts were evaluated through biochemical assays: the xanthine/xanthine oxidase enzymatic assay system, and the NBT/Riboflavine assay. Furthermore, the genotoxic and antigenotoxic activities were tested using the SOS chromotest.

## Method

### Plant material

The leaves of *D*. *gnidium* were collected in the region of Bizerte (latitude: 37.27, height: 22) situated in the North of Tunisia in November 2009. Botanical identification was carried out by Prof. M. Chaieb (Department of Botany, Faculty of Sciences, University of Sfax, Tunisia), according to the flora of Tunisia [12]. A voucher specimen (D9-11-09) has been deposited in the laboratory of Pharmacognosy, Faculty of Pharmacy of Monastir, Tunisia. The leaves were shade dried, powdered and stored (at 25°C under 40% of relative humidity) in a tightly closed container for further use.

### Preparation of plant extracts

In order to obtain an extract enriched with total oligomer flavonoids (TOF), powder (200 g) was macerated in water/acetone mixture (1:2, V/V), during 24 h with continuous stirring. The extract was filtered and the acetone was evaporated under low pressure in order to obtain an aqueous phase. The tannins were partially removed by precipitation with an excess of NaCl during 24 h at 5°C, then we recovered the supernatant. This latter was extracted with ethyl acetate, concentrated and precipitated with an excess of chloroform. The precipitate was separated and yielded the TOF extract which was dissolved in water [19]. Petroleum ether (PE), chloroform (CHl), ethyl acetate (EA) and methanol (MeOH) extracts were obtained by the Soxhlet appartus (6 h). The four types of extract, with different polarities, were concentrated to dryness and the residue was kept at 4°C. The extracts were concentrated to dryness and resuspended in DMSO. These extracts were used at the concentrations of 0.50, 0.75, 1.5 and 3 mg/ml in the Xanthine/xanthine oxydase assay, and at 0.25, 0.5, 1, 2 and 5 mg/ml in the NBT/Riboflavine assay. The doses tested in the SOS Chromotest assay were 50, 250, and 500 μg/assay for petroleum ether and chloroform extracts, 5, 15 and 30 μg/assay with the ethyl acetate and TOF extracts and 50, 100 and 200 μg/assay with the methanol extract.

### Preliminary photochemical analysis

Plant materials were screened for the presence of tannins, flavonoids and coumarins [20]. Two milligrams of each extract were separately dissolved in 2 ml of the adequate solvent. The identification of major chemical groups was carried by thin layer chromatography (TLC) on silica gel 60 F254 Merck (layer thickness 0.25 mm) as follows; for flavonoids, spots were visualized with 1% aluminium chloride solution in methanol under UV (366 nm) [21]. Coumarins were detected under UV (366 nm) thanks to their blue fluorescence which becomes intense after spraying with 10% potassium hydroxide solution in ethanol. The test for tannins was carried out with Fe Cl_3_.

### Bacterial tester strain

*Escherichia coli PQ 37* strain was kindly provided by Prof. M. Quillardet (Institut Pasteur, Paris, France). Frozen permanent copies of the tester strain were prepared and stored at −80°C.

### Determination of total polyphenol and flavonoid content

The polyphenol content of *D. gnidium* was quantified at 25°C by the Folin-Ciocalteau reagent [22,23]. Aliquots of test samples (100 μl) were mixed with 2.0 ml of 2% Na_2_CO_3_ and incubated at room temperature for 2 min. After the addition of 100 μl 50% Folin-Ciocalteau phenol reagent, the reaction tube was further incubated for 30 min at room temperature, and finally absorbance was read at 720 nm. Gallic acid (0.2 mg/ml) was used as a standard. Polyphenol content was expressed according to the following formula:

(1) $$\%Polyphenols=\left(\frac{\left[\frac{DO_{\mathrm{Extarct}}\times 0.2}{DOGallicacid}\right]}{\text{Extract concentration}}\right)\times 100$$

A known volume of each extract was placed in a 10 ml volumetric flask to estimate flavonoid content [24]. After addition of 75 μl of NaNO_2_ (5%), 150 μl of freshly prepared AlCl_3_ (10%), and 500 μl of NaOH (1 N), the volume was adjusted with distilled water until 2.5 ml. After 5 min incubation, the total absorbance was measured at 510 nm. Quercetin (0.05 mg/ml) was used as a standard. Flavonoids content was expressed according to the following formula:

(2) $$\%Flavonoids=\left(\frac{\left[\frac{DO_{\mathrm{Extarct}}\times 0.05}{DO_{\mathrm{Quercitin}}}\right]}{\text{Extract concentration}}\right)\times 100$$

### Determination of tannin content

Extraction of tannin in the sample was achieved by dissolving 5 g of sample in 50 ml of distilled water in a conical flask, allowing the mixture to stand for 30 min with shaking the flask at 10 min intervals, and then centrifuging at 5000 x *g* to obtain a supernatant (tannin extract). The extract was diluted to 100 ml in a standard flask using distilled water.

Five milliliters of the diluted extract and 5 ml of standard tannic acid (0.1 g/l) were measured into two different 50 ml volumetric flasks. One milliliter of Folin-Denis reagent was added to each flask followed by 2.5 ml of saturated sodium carbonate solution. The solutions were made up to the 50 ml mark with distilled water and incubated at room temperature (20-30°C) for 90 min. The absorption of these solutions was measured against that of the reagent blank (containing 5 ml of distilled water in place of extract or standard tannic acid solution) in a Genesys (Wisconsin, USA) spectrophotometer at 760 nm wavelength [25]. Tannin content was calculated in triplicate according to the following formula:

(3) $$\%\;Tanins=\left[\frac{\frac{DO_{\mathrm{Extract}}}{\epsilon }\times 1}{\text{Extract concentration}}\right]\times 100$$

where ϵ; molar extinction coefficient (l.g^−1^. cm^−1^) of tannic acid (= 3.27 L g^−1^ cm^−1^) and l = 1 cm.

### Genotoxicity assay

The SOS chromotest with *Escherichia coli* PQ37 strain was performed according to the procedure described by Quillardet and Hofnung [26]. An overnight culture (16 hours) of *E. coli* PQ37 (100 μL) was added to 5 ml of fresh medium and incubated for 2 h at 37°C. One milliliter of this culture (approximate density 2 x 10^8^ cells/ml) was diluted with 9 ml of fresh Luria broth medium. A fraction of 0.6 ml was transferred into a series of glass test tubes, each containing 20 μl gradual dilutions of the compound to be tested. The mixtures were incubated with shaking for 2 h at 37°C. In order to determine the β-galactosidase (βgal) activity induced by DNA-damaging compound, 2.7 ml of B buffer [Na_2_HPO_4_ (112.7 mM), NaH_2_PO_4_H_2_O (45.8 mM), KCl (10 mM), MgSO_4_·7H_2_O (0.1 mM), sodium dodecyl sulfate (SDS) (3.46 mM), mercaptoethanol 2.7 mL/L, adjusted to pH 7], and 0.6 mL of 0.4% Ortho-Nitrophenyl-β-galactoside (ONPG) solution were added to each tube of one of the sets. Whereas to determine the constitutive alkaline phosphatase (AP) activity, 2.7 ml of P buffer [tris-(hydroxymethyl)-aminomethane (1 M), SDS (3.46 mM) dissolved in distilled water and adjusted to pH 8.8 with HCl], replaced the B buffer, and 0.6 ml of 0.4% p-nitrophenyl phosphate (PNPP) solution was added to each tube of the second set. After incubation, the conversion of ONPG was stopped with 2 ml of 1 M sodium carbonate solution and that of PNPP with 2 ml of 1.5 N sodium hydroxide solution. The absorbance was measured at 420 nm against a blank without bacteria. The induction factor (IF) was calculated as the ratio of R_C_/R_0_, where R_C_ is equal to βgal activity/AP activity determined for the test compound at concentration C, and R_0_ is equal to βgal activity/AP activity in the absence of test compound. The βgal and AP activities were calculated according to the method recommended by Quillardet and Hofnung [26]. Each dose was tested in triplicate.

The doses tested in the SOS Chromotest assay were 50, 250, and 500 μg/assay for petroleum ether and chloroform extracts, 5, 15 and 30 μg/assay with the ethyl acetate and TOF extracts and 50, 100 and 200 μg/assay with the methanol extract.

According to Kevekordes et al. [27], compounds are classified as non-genotoxic if the induction factor (IF) remains <1.5, as marginally genotoxic if the induction factor ranges between 1.5 and 2 and as genotoxic if the IF exceeds 2.

### Antigenotoxicity assay

Inhibition of bacterial genotoxicity was tested in *E. coli* PQ37 strain. Twenty microliters of nitrofurantoin solution (5 μg/assay) was added into tubes with 20 μl of tested concentration of extracts, which were dissolved in DMSO and tested in triplicate [26]. Antigenotoxicity was expressed as percentage inhibition of genotoxicity induced by nitrofurantoin according to the formula:

(4) $$Inhibition\left(\%\right)=100-\left(IF_{1}-IF_{0}/IF_{2}-IF_{0}\right)\times 100$$

where IF_1_ is the induction factor in the presence of the test compound and the mutagen, IF_2_ the induction factor in the absence of the test compound and in the presence of the mutagen, and IF_0_ the induction factor of the untreated cells.

Dose of 5 μg/assay of nitrofurantoin was chosen for the antigenotoxicity studies, since this dose was not toxic and induced a significant SOS response [28].

### Evaluation of xanthine oxidase inhibition effect

The enzyme xanthine oxidase catalyzes the oxidation of xanthine to uric acid. During this reaction, molecular oxygen acts as an electron acceptor, producing superoxide radicals according to the following equation:

(5) $$Xanthine+O_{2}+H_{2}O\to uricacid\;+O_{2}+H_{2}O_{2}$$

Xanthine oxidase activity was evaluated under aerobic condition [29], by the spectrophotometric measurement of the production of uric acid from xanthine. The inhibition of xanthine oxidase activity was followed by measuring the increase of uric acid absorbance at 290 nm as proposed by Cimanga et al. [30], while the superoxide anion scavenging activity was detected spectrophotometrically with the nitrite method described by Oyangagui [31]. The assay mixture consisted of 100 μl of compound test solution, 200 μl xanthine (final concentration 0.1 mM) as the substrate, hydroxylamine (final concentration 0.2 mM), 200 μl EDTA (0.1 mM) and 300 μl distilled water. The reaction was initiated by adding 200 μl xanthine oxidase (11 mU) dissolved in phosphate buffer (KH_2_PO_4_, 0.2 M, pH 7.5). The assay mixture was incubated at 37°C for 30 min. Before measuring the uric acid production at 290 nm, the reaction was stopped by adding 100 μl of 0.58 mM HCL. The absorbance was measured spectrophotometrically against a blank solution prepared as described above, but replacing xanthine oxidase with buffer solution (no production of uric acid). A control solution without test compound was prepared in the same manner as the assay mixture to measure the total uric acid production. The uric acid production was calculated from the differential absorbance. The dose-effect curve for each extract was linearized by regression analysis and used to derive the IC_50_ values. Extracts were used at the concentrations of 0.50, 0.75, 1.5 and 3 mg/ml.

### Determination of superoxide radical scavenging effect

The test implements two principal reactions [32]:

(6) $$2NBTH\to NBT+NBTH_{2}\left(Formazan\right)$$

(7) $$NBTH+O_{2}↔NBT+O_{2}$$

When the riboflavin is photochemically activated, it reacts with the NBT to give NBTH that leads to formazan according to the reaction (a). In presence of oxygen, concentrations of radical species are controlled by the quasi equilibrium (b). Thus, superoxide anions appear indirectly when the test is performed under aerobic conditions. In the presence of an antioxidant that can donate an electron to NBT, the purple color typical of the formazan decays, a change that can be followed spectrophotometrically at 560 nm.

The assay was based on the capacity of the samples to enhance the aerobic photochemical reduction of nitroblue tetrazolium (NBT) in the presence of riboflavine [33]. For all assays, the reaction mixture contained EDTA (6.5 mM), riboflavine (4 μM), NBT (96 μM) and phosphate buffer (51.5 mM, pH 7.4). The volume of tested sample was of 100 μl/assay. The occurrence of superoxide and/or free radicals was indirectly evaluated by the increase in absorbance of formazan at 560 nm, after 30 min of incubation at 30°C from the beginning of illumination [34]. The assay run without any test compound (containing only NBT–riboflavine) was used as the reference. All assays were realized in triplicate.

(8) $$Scavenging\;\%\left(\frac{OD_{\mathrm{controle}}-OD_{\mathrm{sample}}}{OD_{\mathrm{controle}}}\right)\times 100$$

The reference substance (quercetine) was assayed at 0.5, 1 and 2 mg/ml concentrations with three repetitions. Extracts were tested at 0.25, 0.5, 1, 2 and 5 mg/ml in triplicate.

## Results

### Phytochemical study

The TOF, ethyl acetate and methanol extracts showed the presence of various quantities of flavonoids and tannins. Ethyl acetate, chloroform and TOF extracts showed the presence of coumarins. TOF extract showed the highest content of polyphenols, flavonoids and tannins compared to the other tested extracts with respective values of 372.47 equivalent of gallic acid, 494.57 equivalent of quercetin and 163.73 equivalent of tannic acid (Table 1).

**Table 1:** **Quantitative phytochemical screening of extracts from*D. gnidium*leaves**

**Extracts**	**Extract content**		
	**Polyphenols (gallic acid equivalents)**	**Flavonoids (Quercitin equivalents)**	**Tannins (Tannic acid equivalents)**
**Chloroform extract**	104.41	-	-
**Ethyl acetate extract**	227.75	264.57	136.64
**Methanol extract**	157.47	114.57	116
**TOF extract**	372 .47	494.57	163.73

### Genotoxic activity of extracts

In a series of experiments preceding the antimutagenicity studies, it was ascertained that the different amounts of extracts added to the indicator bacteria does not influence their viability.

It was revealed that tested extracts, at the concentrations used, have a very low effect on the induction factor in the SOS chromotest. Based on this, *D.gnidium* tested extracts are evaluated as non genotoxic, except the TOF extract, which can be classified as marginally genotoxic (IF = 2) (Table 2).

**Table 2:** **Genotoxicity induced by extracts from*D. gnidium*leaves**

**Extracts**	**Dose (μg/assay)**	**β-gal (UE)**	**PA (UE)**	**IF**
**Nitrofurantoin**	5	42,18 ± 0,092	11,19 ± 0,0021	23,56
**NC**	0	2,28 ± 0,031	11,27 ± 0,017	1
**Petroleum ether extract**	50	2,26 ± 0,043	13,95 ± 0,017	0,81
	250	1,96 ± 0,002	14,35 ± 0	0,68
	500	2,13 ± 0,012	13,51 ± 0,01	0,79
**Chloroform extract**	50	1,27 ± 0,004	8,13 ± 0,016	0,62
	250	1,52 ± 0,002	8,73 ± 0,007	0,69
	500	3,28 ± 0,015	11,27 ± 0	1,16
**Ethyl acetate extract**	5	0,83 ± 0,001	5,11 ± 0,002	0,73
	15	0,80 ± 0	6,05 ± 0,017	0,60
	30	1,37 ± 0,002	8,15 ± 0,026	0,76
**Methanol extract**	50	0,71 ± 0,041	5,45 ± 0,003	0,59
	100	0,88 ± 0,004	5,92 ± 0,009	0,68
	200	1,31 ± 0,003	6,58 ± 0,017	0,90
**TOF extract**	5	1,88 ± 0	5,89 ± 0,003	2
	15	1,81 ± 0,002	7,98 ± 0,024	1,4
	30	1,35 ± 0,004	6,58 ± 0,024	1,28

β-gal: units of β-galactosidase; AP: units of phosphatase alkaline; IF: induction factor.

Positive control of genotoxicity (nitrofurantoin); NC: negative control (non-treated cells).

### Antigenotoxicity assay

The IFs of nitrofurantoin were determined at various concentrations of *D. gnidium* extracts, and it was revealed that increasing concentrations of TOF, methanol, ethyl acetate and chloroform extracts significantly decreased nitrofurantoin induced genotoxicity (IF).

IFs decreased from 23.56 to 17.37, 6.35 at the dose of 500 μg/ml for petroleum ether and chloroform extracts respectively, to 8.46 and 12.86 at the dose of 30 μg/ml for ethyl acetate and TOF extracts respectively and to 7.50 at the dose of 200 μg/ml for the methanol extract.

The highest inhibition percentages of genotoxicity obtained with the above-mentioned extracts were 47.38% (at a concentration of 30 μg/assay of TOF extract), 71.18% (at a concentration of 200 μg/assay of methanol extract) and 67.95% (at a concentration of 15 μg/assay of ethyl acetate extract). Whereas, petroleum ether extract showed a relatively low efficiency in reducing nitrofuratoin induced genotoxicity, the IF decreased by about 30.5% at the concentration of 50 μg/assay (Table 3).

**Table 3:** **Effect of*D. gnidium*extracts on the genotoxicity induced by nitrofurantoin (5 μg/assay)**

**Extracts**	**Doses (μg/assay)**	**β-gal (UE)**	**PA (UE)**	**IF**	**Inhibition of genotoxicity (%)**
**Nitrofurantoin**	5	42,18 ± 0,092	11,19 ± 0,0021	23,56	-
**NC**	0	3,07 ± 0,024	18,58 ± 0,007	1	-
**Petroleum ether extract**	50	26,21 ± 0,011	9,82 ± 0,019	16,68	30,50*
	250	26,65 ± 0,041	9,61 ± 0,011	17,31	27,70*
	500	27,5 ± 0,019	9,87 ± 0,007	17,37	27,43*
**Chloroform extract**	50	14,31 ± 0	7,05 ± 0	12,29	49,90**
	250	16,18 ± 0,0169	10,29 ± 0	9,53	62,19**
	500	4,66 ± 0,047	4,45 ± 0	6,35	76,28**
**Ethyl acetate extract**	5	8,19 ± 0,019	5,21 ± 0	9,53	62,18**
	15	7,18 ± 0,0183	5,29 ± 0,0028	8,23	67,95**
	30	7,93 ± 0,0127	5,68 ± 0,007	8,46	66,93**
**Methanol extract**	50	14,67 ± 0,001	9,48 ± 0,08	9,38	62,85**
	100	15,98 ± 0,08	10,55 ± 0,04	9,18	63,74**
	200	15,42 ± 0,004	12,464 ± 0,042	7,50	71,18**
**TOF extract**	5	19,73 ± 0,014	8,41 ± 0,009	14,66	39,45*
	15	17,68 ± 0,029	7,04 ± 0,007	15,68	34,92*
	30	14,76 ± 0,009	7,17 ± 0,004	12,87	47,38*

β-gal: units of β-galactosidase; AP: units of phosphatase alkaline; IF: induction factor.

Positive control of genotoxicity (nitrofurantoin); NC: negative control (non-treated cells).

Results were expressed as percentage of inhibition of genotoxicity compared to the positif control (Nitrofurantoin). Values represent the mean ± SD of three separate experiments. The statistical significance of results was evaluated by the Student’s t-test. *P < 0.05, **P < 0.01 means significant difference between positif control and treated sample.

### Evaluation of xanthine oxidase inhibition effect

**Figure 1:**
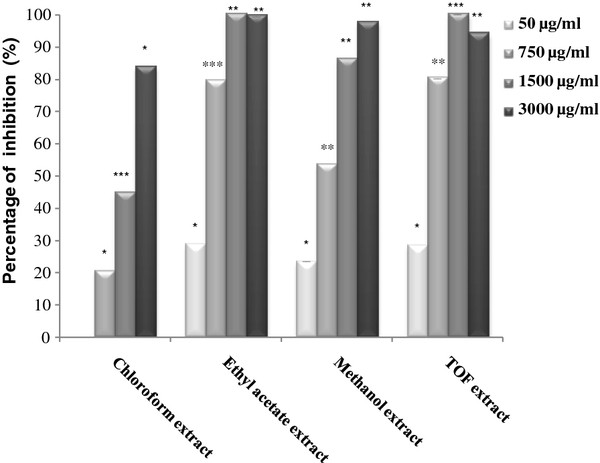
**Inhibition of xanthine oxidase activity by*D. gnidium*leaf extracts.** Results were expressed as percentage of inhibition of uric acid production compared to the control. Values represent the mean ± SD of three separate experiments. The statistical significance of results was evaluated by the Student’s t-test. *P < 0.05, **P < 0.01, ***P < 0.001 means significant difference between control and treated sample.

All the tested *D. gnidium* extracts, exhibited an inhibitory effect on xanthine oxidase activity in a concentration dependent manner. The weakest inhibitory effect was obtained in the presence of petroleum ether extract with a maximal inhibition percentage of 17.66% at 3 mg/ml. TOF and ethyl acetate extracts were the best inhibitors of xanthine oxidase activity with a maximal inhibition percentage of 100% at 1.5 mg/ml (Figure 1). The IC_50_ values of the tested extracts were 1.77, 0.27, 0.50 and 0.28 mg/ml with chloroform, ethyl acetate, methanol and TOF extracts respectively (Table 4).

**Table 4:** ***D. gnidium*leaf extracts IC**_**50**_**for inhibition of xanthine oxidase activity**

**Extracts**	**Inhibition of xanthine oxidase activity IC**_**50**_**(mg/ml)**
**Petroleum ether extract**	**-**
**Chloroform extract**	1.77
**Ethyl acetate extract**	0.27
**Methanol extract**	0.50
**TOF extract**	0.28

### Generation of superoxide anion detected by the non enzymatic NBT/Riboflavine system

**Figure 2:**
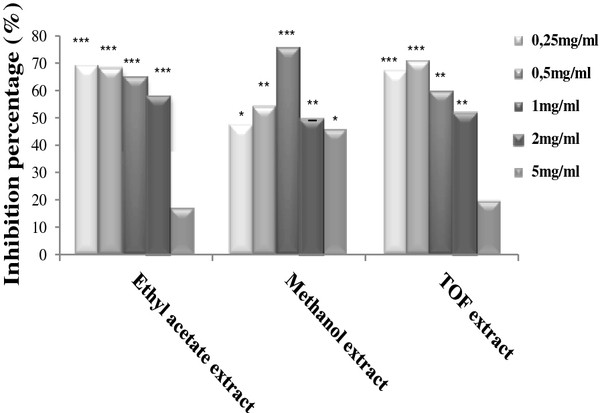
**Antioxidant activity of ethyl acetate, methanol and TOF extracts towards superoxide anion generated by the non enzymatic system NBT/Riboflavine.** Results were expressed as percentage of inhibition of superoxide anion generation compared to the control. Values represent the mean ± SD of three separate experiments. The statistical significance of results was evaluated by the Student’s t-test. *P < 0.05, **P < 0.01, ***P < 0.001 means significant difference between control and treated sample.

**Figure 3:**
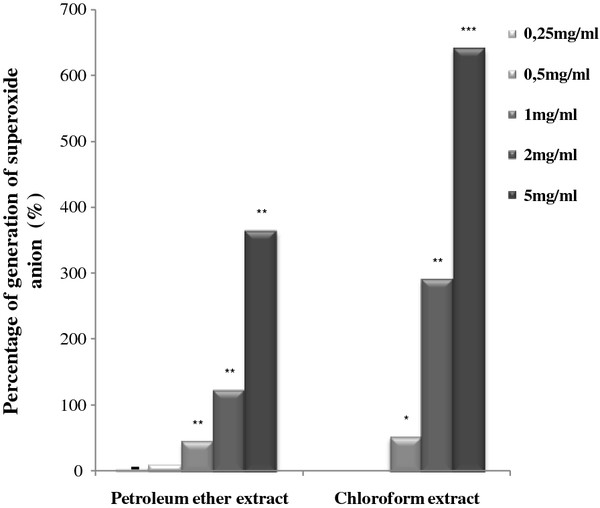
**Pro-oxidant effect of petroleum ether and chloroform extracts towards superoxide anion generated by the non enzymatic system NBT/Riboflavine.** Results were expressed as percentage of superoxide anion generation compared to the control. Values represent the mean ± SD of three separate experiments. The statistical significance of results was evaluated by the Student’s t-test. *P < 0.05, **P < 0.01, ***P < 0.001 means significant difference between control and treated sample.

Methanol extract was the most potent superoxide scavenger in this assay. This extract induced a 57.4% (Figure 2) decrease of NBT photoreduction at a concentration of 0.5 mg/ml and an IC_50_ value of 0.35 mg/ml. Whereas ethyl acetate and TOF extracts exhibited lower scavenging activity with an IC_50_ value of 2.19 mg/ml and 1.62 mg/ml respectively (Table 5). Chloroform, and petroleum ether extracts exhibited a pro-oxidant effect and increased the generation of O_2_^.-^ in dose dependant manner. Chloroform extract seems to be the most important producer of superoxide radical as compared to petroleum ether extract (Figure 3).

**Table 5:** **Superoxide free radicals scavenging effect of extracts from leaves of*D. gnidium***

**Extracts**	**IC**_**50**_**(mg/ml)**
**Petroleum ether extract**	-
**Chloroform extract**	-
**Ethyl acetate extract**	2.19
**Methanol extract**	0.35
**TOF extract**	1.62
**Quercitin**	0.6

- prooxidant effect.

## Discussion

This study is designed to evaluate the antigenotoxic and antioxidant potential of *D. gnidium* extracts employing a variety of *in vitro* methods. The genotoxic and antigenotoxic activity were tested using the SOS chromotest which is a widely used assay for studying genotoxic and antigenotoxic activities of extracts/constituents from medicinal plants. This test employs the error-prone DNA repair pathway of *E. coli* PQ37, also known as the SOS response, a complex regulatory network that is induced by DNA-damaging substances [26,35]. The test involves incubation of the bacteria with the sample under investigation and subsequent determination of β-galactosidase (β-gal) activity. Alkaline phosphatase (AP) activity is also measured as a toxicity control. It was ascertained that different concentrations of *D. gnidium* extracts added to the indicator bacteria did not influence its viability and were non-genotoxic, except the TOF extract which can be classified as marginally genotoxic with an IF value of 2 at the dose of 5 μg/assay in the presence of *E. coli* PQ37. The results indicated that these extracts did not produce DNA lesions which block DNA synthesis, leading to the induction of SOS system. On the other hand, the tested extracts were effective in reducing the IF induced by the nitrofurantoin. In fact, our study showed that all tested extracts were able to reduce the level of DNA damage induced by the nitrofurantoin. These extracts may protect DNA strands from the electrophilic metabolites of the mutagen. Anti-genotoxic activity of the tested extracts may be ascribed to flavonoids [36] coumarins [37], and tannins [38,39] detected in TOF, methanol and ethyl acetate extracts. All these results confirmed our hypothesis that polyphenols contained in the tested extracts are responsible of their antigenotoxicity. In fact, our chemical study let to the identification of apigenin-7-glucoside from methanol and TOF extracts by HPLC analysis (data not shown). Zaabat et al. [40] showed, by SOS chromotest, that this compound prevent the genotoxicity produced by nitrofurantoin. The antigenotoxicity of ethyl acetate and chloroform extracts should be ascribed to the presence of terpenes (beta amyrin acetate) and lignans (dihydrosesamin) we detected in the two mentioned extracts. In fact, Nikolic et al. [41] reported that plant terpenes exhibited antigenotoxic activty. On the other hand Siddique et al. [42] demonstrated that a phenolic lignan (nordihydroguaiaretic acid), possesses an antigenotoxic potential against chlormadinone acetate induced genotoxic damage in mice bone-marrow cells.

We cannot however, exclude the possibility that other compounds with anti-genotoxic properties, participate in the anti-genotoxic effect of chloroform and petroleum ether extracts.

In order to investigate the mechanism by which the *D. gnidium* extracts exert their antigenotoxic effect, the antioxidant activity of the same extracts was evaluated. Use of at least two methods is recommended to assess and compare the antioxidant capacity of a given sample [43]. The present study presents different *in vitro* tests based either on the capacity to inhibit an enzymatic reaction involved in free radicals formation (xanthine oxydase assay) or to scavenge free radicals (superoxide anion). The xanthine oxydase is a flavoprotein which catalyses the oxidation of hypoxanthine to xanthine and generates superoxide anions and uric acid [44]. Consequently, xanthine oxidase is considered to be an important biological generator of superoxide radicals. These and other reactive oxygen species (ROS) contribute to the oxidative stress in the organism and are involved in many pathological processes such as inflammation, atherosclerosis, cancer, aging, etc. [45].

Likewise, a previous study on breast cancer cell line showed that extracts from *D. gnidium* roots have antiproliferative and apoptotic activity against MCF7 cells. Besides, the same study showed a pro-inflammatory effect of *D. gnidium* root extracts at high concentration via prostaglandins E2 (PGE2) and cyclooxygenases (Cox-2) stimulation [46].

In addition our study on erythroleukemia cells showed that extracts from leaves of *D. gnidium* have antiproliferatif effect and induced a perturbation of K562 cell cycle. Chloroform extract inhibited human P-glycoprotein-mediated daunorubicin efflux and enhanced intracellular accumulation of daunorubicin in K562/R7 leukemic cells in a dose dependant manner (data not shown). The inductive effect of *D. gnidium* extracts on the cytotoxicity of MCF7 and K562 cells may also probably be due to its antioxidant properties by perturbing the favorable redox condition and inducing cytotoxicity [47]. Under normal physiological conditions, the endogenous superoxide scavengers in the system protect tissues by neutralizing these radicals. This *in vivo* reaction is simulated in the *in vitro* model so as to use it as an analytical tool to evaluate the ROS scavenging abilities of natural products [48].

The xanthine/xanthine oxidase assay demonstrated that chloroform, methanol, ethyl acetate and TOF extracts were effective inhibitors of xanthine oxidase. In fact, the preliminary chemical study showed that ethyl acetate, methanol and TOF extracts are rich in flavonoids, a group of natural products exhibiting many biological and pharmacological activities. The inhibition of several enzymes by flavonoids has been demonstrated [49,50]. Also, it has been reported that flavonoids inhibit xanthine oxidase [51] and have superoxide scavenging activities. [52,53]. Therefore, it could be a promising remedy for human gout and ischemia by decreasing both uric acid and superoxide concentrations in human tissues [54].

The antioxidant activity of chloroform extract exerted by inhibiting xanthine oxidase activity can be attributed to beta amyrin acetate belonging to terpenes family. The apigenin-7-glucoside detected in both TOF and methanol extracts (data not shown) may participate to their antioxidant activity by inhibiting xanthine oxydase [30]. Luteolin-7-glucoside we identified in methanol extract (data not shown) should also be responsible at least in part,, of the antioxidant capacity this extract as described by Cimanga et al. [30].

Antioxidant capacity of *D. gnidium* extracts was also evaluated by their abilities to scavenge O_2_^.-^ with the non enzymatic NBT/ Riboflavine system. Superoxide radical is known to be very harmful to cellular components as a precursor of more reactive oxygen species [55]. Photochemical reduction of flavins generates O_2_^.-^, which reduces NBT, resulting in the formation of blue formazan [30]. The results indicated that methanol extract was more effective in scavenging O_2_^. -^ than ethyl acetate and TOF extracts. This extract was also more active than the positive control, quercetin, in the assay. It seems that this activity is mostly related to the presence of phenolic compounds such as flavonoids in ethyl acetate, methanol and TOF extracts. The key role of phenolic compounds as scavengers of free radicals is emphasized in several reports [56]. In fact, we have identified, by HPLC, the presence of daphnetin in the methanol and TOF extracts (data not shown). It was reported that this coumarin have a radical scavenging and anti-lipid peroxidation effect [57]. As we have identified the presence of apigenin-7-glucoside in TOF extract and luteolin-7-glucoside in the methanol extract (data not shown), we believe that flavonoids are the most likely candidates among the compounds known to be present in TOF and methanol extracts, for preventing oxidative lesions and providing antigenotoxic effect [58,59].

Chloroform and petroleum ether extracts showed a pro-oxidant effect with the non enzymatic system NBT/Riboflavin. In fact, several studies showing controversial results of exogenous antioxidants (including polyphenols) debating that the type, dosage and matrix of these antioxidants may be determining factors impacting the balance between beneficial and deleterious effects of these natural compounds [60]. There are also some proofs that they act as pro-oxidants, under certain conditions, such as high doses or the presence of metal ions [61-63]. The antioxidant or pro-oxidant activity intimately depends on their concentration [60]. Besiedes, some of the most abundant phenolic acids present in foods were reported to act as pro-oxidants: caffeic, chlorogenic, coumaric and ferulic acids [64-68].

## Conclusion

The present study has demonstrated that some *D. gnidium* extracts possess potent antioxidant and antigenotoxic activities, which could be derived from compounds such as flavonoids and phenols. The antigenotoxic activity could be ascribed, at least in part, to their antioxidant properties but we cannot exclude other additionally mechanisms. However, further work is required to determine mechanisms involved in the antioxidant and antigenotoxic effects. In addition, *in vivo* evidence and identification of active phenolics involved is needed.

### Statistical analysis

Data are expressed as the arithmetic means ± SD of 3 separate experiments. The statistical significance of results was evaluated by the Student’s t-test, with probability values of 0.05 being considered as significant.

## Competing interests

The authors declare that they have no competing interests.

## Authors’ contributions

FC carried out SOS chromotest assay, NBT/riboflavin assay and Xanthine oxidase assay. JB carried out polyphenols, flavonoids and tannins quantification and contribute to the SOS Chromotest assay. AL carried out extract preparation. AN carried out photochemical analysis. SK prepared *E.coli* 37 and solutions which have been used for SOS chromotest, NBT/Riboflavine and xanthine oxidase assays. KG assisted with *Daphne gnidium* extraction and study design and interpretation. LCG helped conceive the study and helped in the preparation of the manuscript. All authors read and approved the final manuscript.

## Pre-publication history

The pre-publication history for this paper can be accessed here:

http://www.biomedcentral.com/1472-6882/12/153/prepub
